# Gaze and Arrows: The Effect of Element Orientation on Apparent Motion is Modulated by Attention

**DOI:** 10.3390/vision1030021

**Published:** 2017-08-22

**Authors:** Rossana Actis-Grosso, Paola Ricciardelli

**Affiliations:** 1 Dipartimento di Psicologia, Università degli Studi di Milano-Bicocca, 20126 Milano, Italy; 2 Centro di Neuroscienze di Milano (MI), 20126 Milano, Italy

**Keywords:** apparent motion, correspondence problem, attention based motion perception, automatic orienting of attention, gaze-mediated orienting of attention

## Abstract

In two experiments we investigated whether stimuli that elicit automatic orienting of attention (i.e., arrow or averted gaze) could drive apparent motion perception in one of two possible directions, modulating the effect of a low-level property (the orientation of elements along the motion direction). To this end, the competing motion paradigm was used, in which at time 1, a stimulus appears in the center of the display, and at time 2, two other stimuli appear in different spatial locations. Three kinds of stimuli with eight possible orientations were used in separate blocks; (1) a line; (2) an arrow; and, (3) an averted gaze. First, since the three stimuli present in the display at time 2 should be perceived to be located at the same distance (i.e., equidistant), the threshold for perceived equidistance was calculated for each participant and then used as the customized inter-stimulus distance. Participants were asked to press the button corresponding to the direction of the perceived motion. Results show a preference for collinear motion (motions between elements oriented along the motion direction), with a higher percentage of responses for gaze and arrow stimuli. In Experiment 1, a difference between gaze- and arrow-stimuli was observed. Apparent motion was seen towards the collinear position more often for gaze than for arrow when the stimulus was pointing to the vertical directions, while the opposite was true when the stimulus was pointing to the horizontal directions. In Experiment 2, where the lightness contrast between the gaze and the background was reduced, no difference between gaze- and arrow-stimuli emerged. We interpret our results as due to the social and biological value of gaze, which solved a possible ambiguity between gaze direction and the directions conveyed by the figural properties of the contrasted background in Experiment 1. These findings are consistent with the idea that stimuli known to automatically orient visual attention modulate motion perception.

## 1. Introduction

### 1.1. The Correspondence Problem

**Figure 1:**
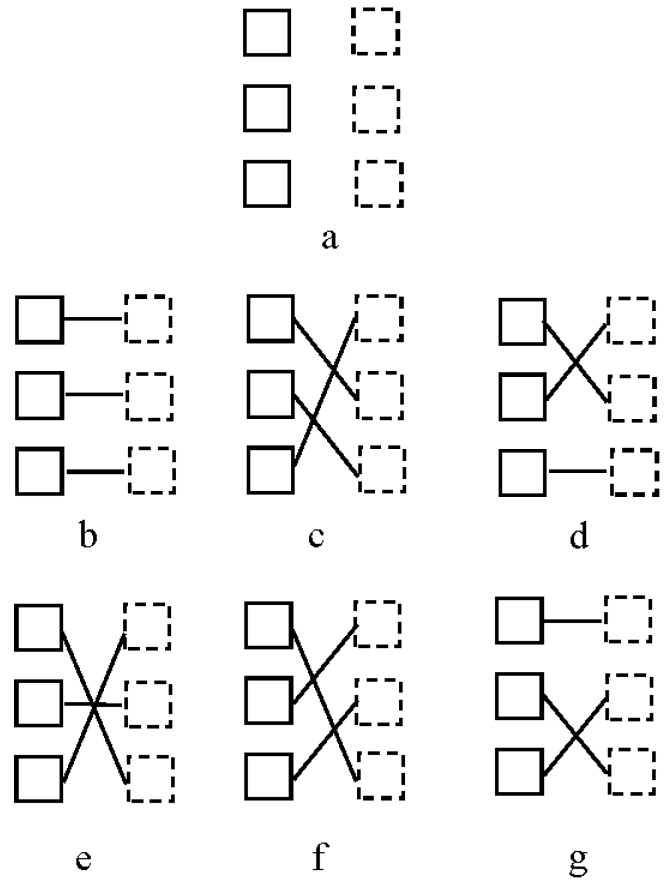
(**a**) An example of an apparent motion display. Outline squares represent element positions in Frame 1; dashed squares represent element positions in Frame 2. (**b**–**g**) Possible motion correspondence solutions for display (**a**): solid lines indicate the assigned motion correspondence matches. Solution (**b**) is the one generated by the human visual system. Adapted from [5].

The visual system maintains stable object representations, although the information represented in the proximal stimulus cannot, by itself, completely determine the nature of the distal stimulus. Retinal stimulation, in fact, cannot unequivocally determine the perceptual outcome corresponding to the physical world. This uncertainty can also result because the information available from local measurements of the proximal stimulus is consistent with a large number of different global interpretations. One example of this is the so-called *motion correspondence problem* (e.g., [1,2,3]). This is better understood when considering an apparent motion display. To generate apparent motion, the human visual system must identify an element in a position in one image (Frame 1) and another element in a different position in the next image (Frame 2), as constitutes different glimpses of the same moving element. The correspondence problem then becomes one of establishing a match between parts of one frame and their counterparts in a subsequent frame that represent the same object at a later time (see Figure 1).

This problem has been studied for at least a century (e.g., [3,4,5,6]), and spatio-temporal parameters (such as optimal spatial and temporal intervals between presentations of image elements) have been psychophysically quantified in many experiments with human subjects (e.g., [7,8,9,10]) and animals (e.g., [11,12,13,14]). However, less attention has been paid to the figural properties, such as geometrical relations and topological properties of the image elements, which are crucial to the correspondence process. Since the pioneering work of Fernberger [15] and Orlansky [16], who report effects of similarity (line width) between line segments and the perception of apparent motion, a substantial but contradictory literature emerged on the role of figural properties on the correspondence problem. Seminal papers by Kolers and Pomerantz [17] and Navon [18] showed that figural identity does not have any effect on determining the type of motion experienced. However, in 1990 Werkhoven, Snippe and Koenderink [19] reported the crucial role of a particular figural property as the orientation of line elements relative to motion direction. In their work, motion perception between elements oriented along the motion direction (e.g., a vertical line moving along a vertical direction) dominates motion perception between elements oriented perpendicularly to motion direction (e.g., a vertical line moving along a horizontal direction).

### 1.2. The Role of Attention in Motion Perception

The aim of this study was to investigate the role of particular figural properties, namely the aspect of form combined with geometrical relations, in solving the correspondence problem. Our idea stemmed from the role of attention in motion perception, which is often crucial. For example, when a stimulus contained two components moving in opposite directions, the motion is seen in the direction, which fell under the attentive control of the observer (i.e., attentive tracking; e.g., [20,21]). Many neurons in the human primary visual cortex are sensitive to the direction of motion [22], and attention might act by selecting one or the other of these low level motion responses. However, it has been demonstrated [23] that the perception of motion during attentive tracking can arise independently of low-level motion responses, and may be derived from the internal signals that move the focus of attention. This phenomenon, first described in 1912 by Wertheimer [24], is now often referred to as attention-based apparent motion (e.g., [25]), or attention-based motion in the case of a continuous stimulus [23].

Cavanagh and Mather [26] introduced a distinction between passive and active motion processes. In their view, attention based motion perception, as well as attentive tracking, are phenomena that fall into the category of active perception. In active perception, it is the observer’s conscious effort (i.e., internal signals) that drives perception, resulting in the perception of motion in one of two possible directions. In the case of passive motion perception, on the other hand, it suffices to open the eyes, and if there is an unambiguous displacement of an object, it will be perceived as moving in a particular direction.

### 1.3. Stimuli that Trigger Reflexive Attention Shifts and Their Possible Role in Apparent Motion Perception

The question arises regarding whether external stimuli that elicit automatic orienting of attention could drive apparent motion perception in one of two possible directions. Regarding the stimuli that elicit the automatic orienting of attention, the study of visuospatial attention mechanisms has focused on trying to identify the stimuli that are potentially able to elicit reflexive attention shifts by means of the spatial-cueing paradigm [27]. In this paradigm, a peripheral target requiring a response is preceded by a cue with a spatially congruent or incongruent spatial vector, aimed at testing whether performance is enhanced when the target appears at a location in which attention has been oriented. Evidence has been provided showing that not only peripheral abrupt onset cues [28], but also arrows (i.e., a central cue) can trigger reflexive attention shifts, even when they are uninformative with respect to target location, thus suggesting that arrow-mediated orienting can be defined as automatic [29,30,31]. Furthermore, it has also been shown that participants tend to shift attention following the direction signaled by the gaze of a face presented at fixation (e.g., [32,33,34,35,36,37,38]), a phenomenon known as gaze-mediated orienting (or joint attention).

In all of the studies cited above external stimuli (i.e., directional cues such as arrows and averted gaze) have been used to cue a spatial location. One could wonder what would happen if attentional directional cues were not used as cues, but as the moving stimuli in an ambiguous motion paradigm. Would directional cues known to engage attention orienting mechanisms (such as pointing arrows and averted gazes), have also different effects in solving the correspondence problem? We hypothesized that the stimuli known to automatically trigger attention shifts in a direction should also drive the perception of motion in the direction to which they are pointing when they move, in a sort of “passive tracking” fashion. This is because these signals do not require an active or conscious effort in orienting attention, and for this reason, according to Cavanagh and Mather [26], they cannot fall into the category of active perception. For example, one could expect that if an averted gaze—which automatically drives attention ([32]; for a review see [34])—is used as a moving stimulus in an ambiguous motion paradigm, the ambiguity could be solved more easily in the direction indicated by the gaze itself as compared to that of a line oriented along the motion direction [19]. In this way, we are combining low-level figural properties, such as element orientation, with higher-level figural properties, such as the aspect of form (i.e., being an arrow or an averted gaze). In other words, we expected that directional cues (which automatically drive attention) should play an important role in solving the correspondence problem, modulating the effect of low-level properties (such as the orientation of elements along the motion direction).

### 1.4. Rationale for Experiment 1: Using Stimuli that Elicit Automatic Orienting of Attention as Moving Stimuli in an Ambiguous Motion Paradigm

Our working hypothesis was thus that attention orienting mechanisms could differently influence the perception of motion when mediated by stimuli, which are all oriented relative to motion path, but have different saliency in terms of their adaptive or symbolic nature. 

To test this hypothesis we compared three different kinds of stimuli: (1) an oriented line; (2) an arrow (commonly used to orient visual attention, [39]); and, (3) an averted gaze. The choice to use also an arrow was motivated by different reasons. On the one hand, an arrow, as an averted gaze, automatically drives attention in a given direction [31,40], but it also shares some figural properties with an averted gaze and some others with a simple line. As an averted gaze, an arrow points towards a given direction, whereas a line is virtually oriented towards two opposite directions. As a line, an arrow is always oriented, whereas a gaze could also be non-oriented (i.e., looking straight ahead); again, as for a line, the orientation of an arrow could be extremely precise, whereas a gaze can be broadly oriented in a given direction (e.g., to the left or to the right, although people when asked to discriminate gaze direction are very precise, see [41,42]). On the other hand, the human gaze has a crucial role in establishing social relationships (e.g., [43]), and it has a social and emotional meaning, which goes beyond its simple orientation. For example, a gaze, which is both averted and emotionally scared, automatically drives attention in a way that has a stronger adaptive nature than an arrow pointing in the same direction. For this reason, we expected to find a difference in the modulation of the effect of the arrow, and the oriented gaze on the perceived direction of an ambiguous motion. To this end, we used a real photograph of a gaze, to preserve as much as possible in a controlled environment the ecology of the seen gaze.

To test whether apparent motion perception would be differently affected by the direction conveyed by the three different kinds of stimuli, a competing motion paradigm was used [3]. In this paradigm, at time 1, a stimulus appears in the center of the display and at time 2, two “test” stimuli appear in different spatial locations, such as one to the left and one to the right. The direction of motion reported by participants, either left or right, indicates which stimulus “wins”. Because the participant has to make a choice between two stimuli, the selection of one over the other should indicate that this stimulus is seen as the same object as the stimulus at time 1, thus indicating the direction of motion [44]. The three stimuli should be perceptually equidistant (i.e., perceived located at the same distance), because it is known [24] that the motion correspondence problem is always solved in favor of the nearest stimulus. Therefore, given that our aim was to test the role of directional cues, in a preliminary session (static session) the threshold for subjective equidistance has to be calculated for each participant, so to use it as inter-stimulus distance in the competing motion paradigm (dynamic session).

## 2. Experiment 1

### 2.1. Methods

#### 2.1.1. Participants and Apparatus

Twenty participants voluntarily took part in this experiment (age range: 19–32 years old; mean age: 25.7; female: 11). They were all naïve as to the experimental purposes and, prior to participation, they signed an informed consent. They all had normal or corrected-to-normal vision, and were right-handed. The experimental protocol was approved by the local committee and carried out in agreement with legal requirements and international norms in accordance (the Declaration of Helsinki). Each participant completed two experiment sessions, each performed on different days. Stimulus presentation and data recording were controlled by means of a PC (Personal Computer) Pentium 4 computer, connected to a 21 in. monitor with a resolution of 1600 × 1200 pixels and a refresh rate of 100 Hz. E-Prime 2.0 (Psychology Software Tools, Inc., Pittsburgh, PA, USA) was used for stimuli presentation and data recording.

In both sessions, participants sat in a dimly room in front of a PC monitor, with a chinrest maintaining their eyes at a distance of 57 cm from the monitor where visual stimuli were displayed. During the first session, a black paper board with an oval shape was placed around the PC screen as a mask. Such a mask was aimed at preventing participants from using reference points during the experiment running.

#### 2.1.2. Stimuli, Procedure and Design

For both the first and the second session, the following types of stimuli were used: (1) a black segment (116 × 5 pixels, corresponding to 4.2 deg of visual angle); (2) a black arrow (116 × 5 pixels, corresponding to 4.2 deg of visual angle; endpoint: two segments of 1 deg of visual angle each). The endpoint of the arrow was always oriented towards the outer; and, (3) a rectangular black and white picture of a human gaze (185 × 50 pixels). Within this picture, the two eyes were naturally aligned, with an inter-pupillary distance of 42 mm (i.e., 116 pixels, corresponding to 4.2 deg of visual angle), and looking at one of eight different directions. The experiment was divided in two different sessions that will be referred to as “static” and “dynamic”, respectively. Different procedures were used for the two sessions, which will be separately described below. 

**Figure 2:**
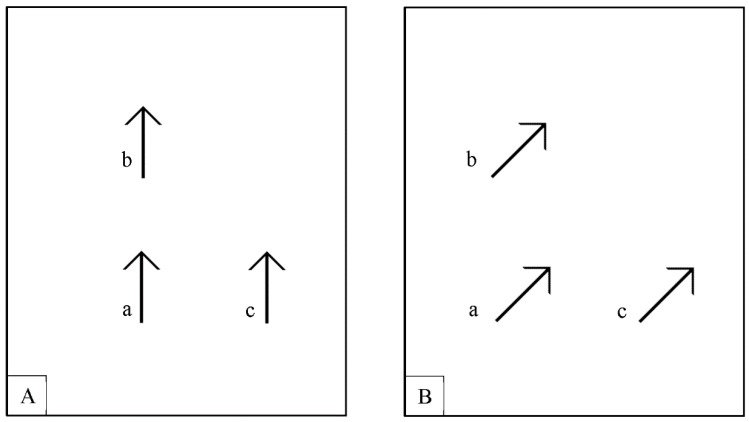
(**A**,**B**) Two examples of the display in Static Session, where “b” arrows are in a position which is collinear to the position of the central arrows, here denoted with “a”. The figure is not drawn to scale.

*Static session.* During the static session, for each stimulus type, three stimuli were simultaneously displayed on the screen. These stimuli were arranged as follows (see Figure 2): one was always presented at the center of the PC monitor (i.e., central stimulus, labelled as “a” in Figure 2), whereas the other two stimuli (labelled as “b” and “c” in Figure 2) were in the collinear position of the central stimulus (stimulus “b”), and at one of the other seven positions around the central stimulus (stimulus “c”). The initial distance between the three stimuli was randomly chosen from an interval comprised between 5.2 and 2.2 deg of visual angle.

**Figure 3:**
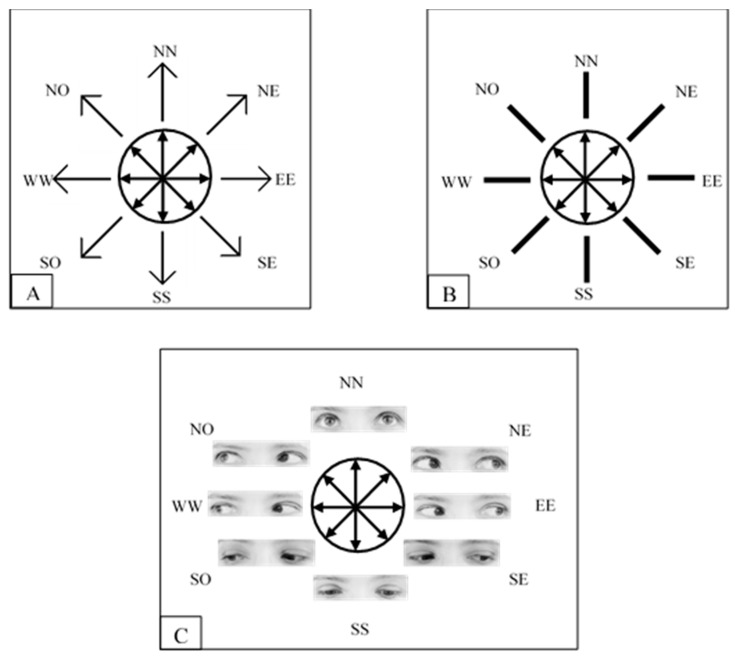
(**A**–**C**) The three types of stimuli used in Experiment 1. Each panel displays the eight directions conveyed by the different stimuli and the eight possible positions of stimulus presentation. The figure is not drawn to scale.

Stimulus positions corresponded to a circular array having its center on the middle point of the central stimulus (see Figure 3). These positions will be referred to as the cardinal points North-North, North-East, East-East, South-East, South-South, South-West, West-West, North-West (NN, NE, EE, SE, SS, SW, WW, NW, respectively). In the line condition given that the initial orientation was ambiguous, since it could indicate one direction or its opposite (e.g., either NN or SS), half of the time it was labelled as one direction (e.g., NN) and the other half as the opposite (e.g., SS).

The static session aimed at determining the threshold of subjective equidistance for each participant by means of the adjustment method. To this aim, the participants were requested to move one of the two peripheral stimuli as to make its distance from the central stimulus equal to the distance between the other lateral stimulus and the central one (see Figure 2). The participants pressed the “P” key with their right hand to move the stimulus to the right, and the “Q” key with their left hand to move the stimulus to the left. Half of the time the stimulus to be adjusted was the collinear stimulus, and the other half the non-collinear one. The threshold for perceived equidistance for each type of stimulus was calculated in three different blocks (i.e., lines, arrows and gaze). The presentation order of the three blocks was counterbalanced between participants: one participant was presented with the presentation order Lines-Arrows-Gaze (LAG), the other one LGA, and so on for the 6 possible combinations. In each block, the threshold for perceived equidistance was calculated for the eight possible spatial positions for each orientation of the central stimulus. Each of the 56 possible combinations was repeated twice, for a total of 112 trials per block, presented in a random sequence. Then each participant performed 336 trials. The static session took about 1 h and 30 min. Participants were allowed to take a break after about 45 min. 

*Dynamic session*. In the dynamic session, the same line, arrow, and gaze used in the static session were used as stimuli. As for the static session, each type of stimulus was presented in separate blocks, resulting in three blocks in total. The presentation order of the three blocks was counterbalanced across participants as in the static session. For each block, each trial started with the appearance of a stimulus at the center of the screen on a light background. This stimulus lasted for 500 ms. After 250 ms, two stimuli, identical to the first stimulus, were presented at two of the eight positions as those used for the static session.

Since we expected that the correspondence problem should be solved in the direction indicated by the orientation of the stimulus presented at t1, one of the two positions was always “competing” with the direction indicated by the stimulus presented first. This means that, if the first stimulus was an arrow pointing to NN, then one of the two stimuli presented after 250 ms was always presented at position NN (elements oriented along the direction of motion, hereafter defined as collinear elements), whereas the competing stimulus could be at any of the seven remaining positions around the central stimulus.

**Figure 4:**
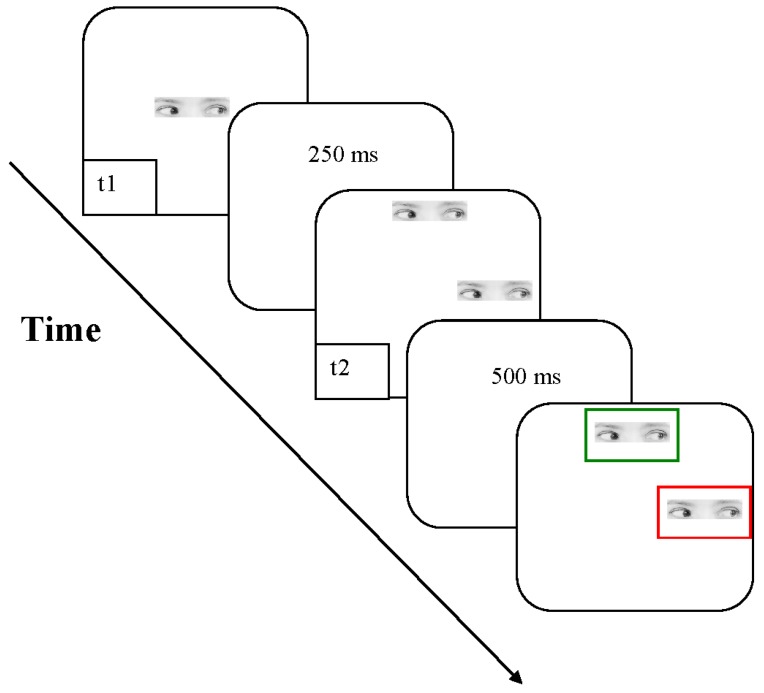
A schematic representation of the sequence of events in Experiment 1, with gaze as stimuli. Please note that stimuli presented in t2 are always oriented in the same direction as stimulus in t1. The figure is not drawn to scale.

For each participant, the distance between the stimulus presented at t1 and the two stimuli presented at t2 differed and corresponded to his/her perceived equidistance as calculated in the static session. After 500 ms from the appearance of these two stimuli, two rectangular frames—one red and the other green—were superimposed on these two stimuli (see Figure 4). Color frame was randomly assigned to the two test stimuli. The dimension of this frame was 214 × 90 pixels. The frame indicated to the participants the key to press. The two response keys (P and K) were labelled with a red and green mark. Participants were instructed to press the button marked green if they had perceived the motion towards the stimulus with the green frame, and to press the button marked red if they had perceived the motion towards the stimulus with the red frame. They were asked to be as accurate as possible. No response times were recorded.

The experimental design included three factors: Orientation (8 levels) × Position (7 levels) × Type of stimuli (3 levels). Each trial was repeated twice, making 336 trials in total. Ten trials were run before each session to familiarize the participants with the experimental task. The second session took about 45 min.

### 2.2. Results

Values for the subjective equidistance threshold were automatically calculated for each participant and used by the script to set distances—for the same participant—between the central and the lateral stimuli in the dynamic session. Data from the first session were not analyzed further. The dependent measure for the dynamic session was the collinearity index (CI). Such an index was calculated as the percentage of responses in which the participants selected the collinear stimulus as that towards which the apparent motion was seen. The collinear stimulus is considered as the stimulus presented in the position correspondent to the direction conveyed by stimulus orientation (See Figure 2). In other words, a CI of 100 would indicate that the central stimulus was always perceived as moving towards the position that was collinear with its orientation, whereas a CI of 0 would indicate that the central stimulus was always perceived as moving towards the non-collinear position. Thus, a CI of 50%, corresponding to chance level, would indicate uncertainty. In order to determine whether participants’ responses significantly differ from those that would have been obtained at chance level, a one sample *t*-test was performed against 50 for the three types of stimulus (all *ps* < 0.05). However, the *t*-test performed against 50 for each type of stimulus in each orientation proved non significant for the line in orientations NN, EE, SW, and NW, respectively; for the arrow in the orientation NN, and for the eyes in the orientations EE and WW (all *ps* > 0.05).

The ANOVA performed on the CI revealed a significant main effect of factor “Type of Stimulus” (F(2,38) = 6.812, *p* = 0.003, η^2^ = 0.263). In particular, the participants tended to choose the collinear stimulus in a greater proportion when the stimulus was an arrow than when it was a line, and when the stimulus was a gaze than when it was either an arrow or a line (CI mean values = 74.42, 68.5 and 62.55, for gaze, arrow, and line, respectively, post-hoc *t*-tests were significant for all differences: *t* = 2.385, *p* = 0.018 for the difference between line and arrows, *t* = 5.193, *p* < 0.001 for the difference between line and gaze and *t* = 3.094, *p* = 0.002 for the difference between arrow and gaze).

**Figure 5:**
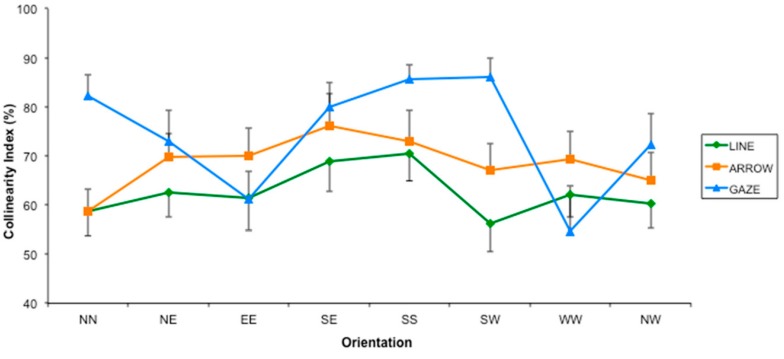
Collinearity Index (indicating the percentage of responses towards the collinear direction) for the three types of stimuli as a function of the eight possible orientations (Experiment 1). Error bars indicate the Standard Error. North-North, North-East, East-East, South-East, South-South, South-West, West-West, North-West (NN, NE, EE, SE, SS, SW, WW, NW, respectively).

Interestingly, this analysis also revealed a significant interaction “Type of Stimulus by Orientation” (F(14,266) = 3.562, *p* < 0.001, η^2^ = 0.157). As shown in Figure 5, the interaction was mainly due to the stimulus “gaze”, which differently modulated the effect of orientation on the correspondence solution. On the one hand, the pattern of the CI found for the arrow followed the one for the line, although its value was higher. On the other hand, the pattern of the CI found for the gaze was different. This was because for some orientations, the CI for the gaze was the same as the CI for the line, thus lower than the arrow itself, whereas for the other orientations the CI for gaze was higher than both the CI for the line and for the arrow.

**Figure 6:**
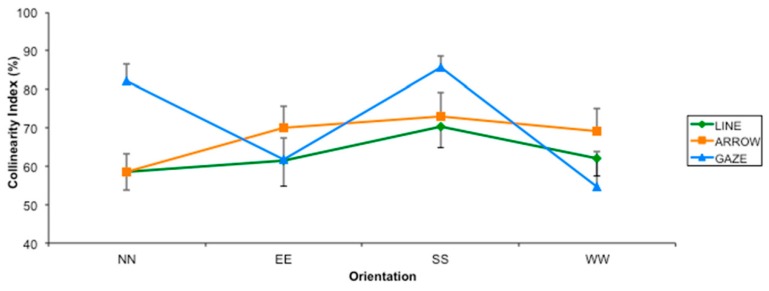
Collinearity Index (indicating the percentage of responses towards the collinear direction) for the three types of stimuli as a function of the four principal orientations (i.e., vertical, NN and SS, and horizontal, EE and WW) in Experiment 1. Error bars indicate the Standard Error.

In particular, if we compare the patterns of responses of gaze and arrows, the proportion of responses towards the collinear is higher for gaze when the direction indicated by the orientation is on the vertical axes, whereas it is higher for arrows when the direction indicated by the orientation is on the horizontal axes (all *ps* < 0.5, with the exception of direction EE, *t* = 2.882, *p* = 0.08). The only exception was the SW direction, which showed a significant difference between gaze and arrow (*t* = 2.634, *p* = 0.016). We could better appreciate this difference limiting our analysis to the four orthogonal directions (see Figure 6). As it is apparent in Figure 6, data for horizontal directions (EE and WW) were similar for the three types of stimuli, whereas the data for vertical directions (NN and SS) were considerably higher for the gaze stimuli.

### 2.3. Discussion

The first important result of this experiment is that directional cues known to engage attention orienting mechanisms do have an effect in solving the correspondence problem, as indicated by a higher CI for both arrow and gaze, as compared to lines.

In Experiment 1, we found that, as expected, apparent motion perception was differently modulated by different types of stimulus. Specifically, apparent motion was seen towards the position in which the stimulus was oriented in a higher proportion when the stimulus was an arrow than when it was a line, and when it was a gaze than when it was either an arrow or a line.

Another interesting result showed a difference between gaze and arrow in the modulation of apparent motion across orientation. In particular, apparent motion was seen towards the position in which the stimulus was oriented in a higher proportion for gaze than for arrow when the stimulus was pointing to the vertical directions, but in a higher proportion for arrow than for gaze when the stimulus was pointing to the horizontal directions. The difference between gaze and arrow may be due to the fact that for the gaze stimuli, we presented only the eye region, which could induce to see them within a letter box window, perceived as a horizontal bar (i.e., an oriented line with a higher width than that of the line used as stimulus in this experiment). This may have put in conflict the direction conveyed by the figural properties of the letter box with the direction conveyed by the gaze. When the gaze was pointing towards a direction which was *orthogonal* to that suggested by the figural properties of the bar (i.e., SS and NN, which correspond to vertical directions), this information might be considered salient, given that was somehow suggesting an important behavioral information which was not suggested by the figural property of the horizontal bar. In such situations, the fact that the gaze has also an important biological and social value might be the reason for “winning the competition”. On the contrary, when the gaze was pointing towards a direction, which was consistent with the figural properties of the letter box (i.e., EE and WW, which correspond to the horizontal directions), the information given by the gaze might be considered redundant, and thus ignored.

To test this possibility we designed a control experiment, where, if our hypothesis was correct, we would expect a different pattern of results between the gaze and the other two types of stimuli.

## 3. Experiment 2

**Figure 7:**
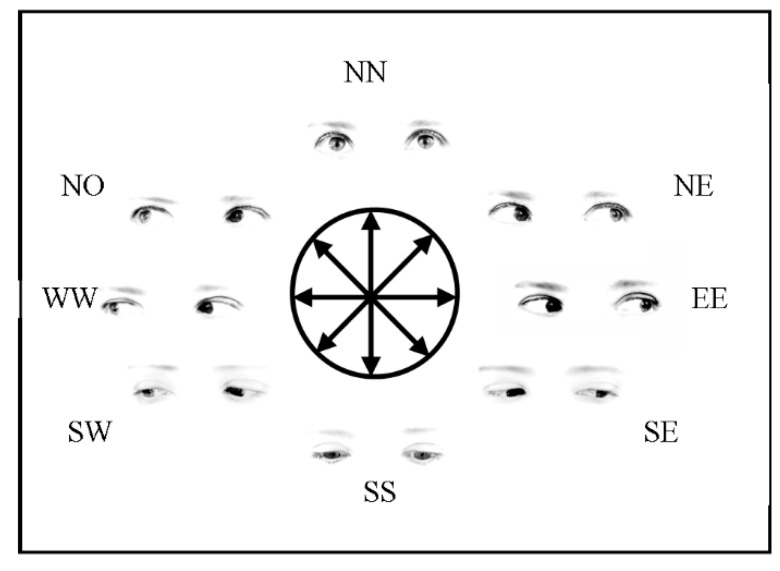
The stimulus type “gaze” used in Experiment 2, displaying the eight directions conveyed by the different stimuli and the eight possible positions of stimulus presentation. The other stimulus types (i.e., “line” and “arrow”) were the same as in Experiment 1.

In Experiment 2 we reduced the conflict between the directional information conveyed by the figural properties of the letter box in which the gaze appeared with the directional information conveyed by the gaze itself. To this end, we reduced the lightness contrast between the gaze and the background (so that no frame was visible around the gaze-stimuli), but preserving both the ecological validity of the gaze and its directional meaning (see Figure 7).

In particular, for the gaze stimuli we expect that for those positions in which in Experiment 1 the conflict between figural (i.e., the frame around the stimuli) and directional (i.e., the averted gaze) properties was maximum, namely positions SS and NN, the correspondence problem should be solved less often towards the collinear gaze-stimulus as compared to Experiment 1.

### 3.1. Methods

#### 3.1.1. Participants

Twenty-two new participants voluntarily took part in this experiment (10 females, age range: 19–32 years old; mean age: 23.3).

#### 3.1.2. Apparatus, Stimulus, and Procedure

They were the same as in Experiment 1, except that now we adjusted the lightness contrast of the gaze stimuli by means of Adobe Photoshop so that no frame was visible around the stimuli (see Figure 7).

### 3.2. Results and Discussion

As in Experiment 1, the values for subjective equidistance threshold were automatically calculated for each participant and used by the script to set distances—for the same participant—between the central and the lateral stimuli in the dynamic session. Data from the first session were not further analyzed. The dependent measure for the dynamic session was the collinearity index (CI), as calculated in Experiment 1. As before, in order to determine whether the participants’ responses significantly differed from those that would have been obtained at a chance level, a one sample *t*-test was performed against 50 for the three types of stimulus and each type of stimulus in each orientation (all *ps* < 0.001 with the exceptions of line in orientations SE, SS and SW, with all *ps* < 0.05).

The ANOVA performed on the CI revealed a significant main effect of factor “Type of Stimulus” (F(2,38) = 17.432, *p* < 0.001, η^2^ = 0.478). In particular, the participants tended to choose the collinear stimulus in a greater proportion when the stimulus was either a gaze or an arrow than when it was a line (CI mean values = 78.9 and 74.8 vs. 64, for arrow, gaze and line respectively, post-hoc *t*-tests: *t* = 8.704, *p* < 0.001 for the difference between line and arrows, *t* = 6.324, *p* < 0.001 for the difference between line and gaze and *t* = 2.546, *p* = 0.126 for the difference between arrow and gaze).

**Figure 8:**
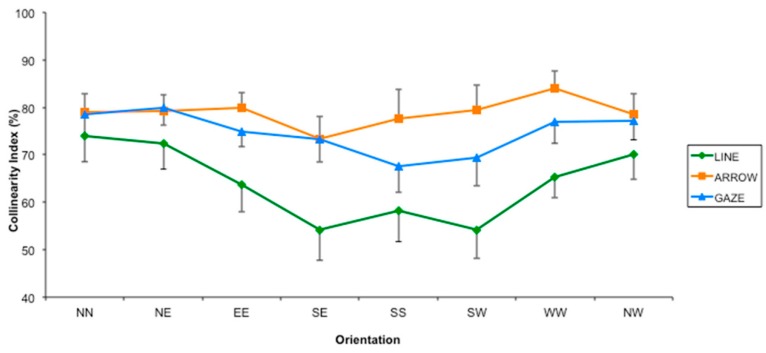
Collinearity Index (indicating the percentage of responses towards the collinear direction) for the three types of stimuli as a function of the eight possible orientations in Experiment 2. Error bars indicate the Standard Error.

The results confirmed our hypothesis, given that the directional information conveyed by gaze was no more differently modulated by the factor “Orientation” (see Figure 8). In particular, both the main effect of factor “Orientation” and the interaction “Type of Stimulus by Orientation” were not significant (F(7, 69,93) = 2.67; *p* > 0.05 and F(14, 146,267) = 1.922; *p* > 0.05, respectively). Thus, reducing the conflict between the directional information conveyed by the gaze with the directional information conveyed by the letter box, in which the gaze appeared in Experiment 1 was enough to cancel out the difference between gaze and arrow found in it. This result is in line with our hypothesis that gaze specificity was due to the crucial role of gaze in solving a possible ambiguity between vertical direction (conveyed by the gaze itself) and the horizontal directions conveyed by the figural properties of the letter box in which the gaze was inserted. Unlike Experiment 1, we also found a higher (although non significant) CI value for arrows than for gazes. This was probably due to the reduced contrast of gaze with the background in this Experiment.

## 4. General Discussion

In the present study, we demonstrate that the directional cues known to engage attention orienting mechanisms do have an effect in solving the correspondence problem. 

Here, we reported that this effect is enhanced when arrows and gaze directions are used as stimuli in the motion correspondence paradigm. Previous results showed that the orientation relative to motion direction is an important feature of visual elements [19], so that in a competing motion paradigm there is a dominance of motion path when visual elements move along their orientation axis. Our results extend previous results by showing that also the form of visual elements is an important feature in driving apparent motion perception along a given direction. Specifically, this happens when the form of elements (i.e., averted gaze and arrows) contains directional information that automatically triggers attention orienting mechanisms.

Previous results have been interpreted in terms of both receptive field form, elongated in the motion direction [19], and spatial integration in the direction of motion [45]. Our results link this low-level interpretation with both attentive tracking and attention-based apparent motion [23]. The novelty of our study consists in demonstrating that attentive tracking could take place also when it is not derived by internal signals that moves the focus of attention (i.e., endogenous orienting), but it is instead triggered by stimuli, which caused an attention shift in the direction to which these stimuli are pointing when they move (i.e., exogenous orienting). Thus, as we hypothesized in the introduction, our stimuli give raise to a “passive tracking”, where the stimulus-driven orientation of attention played a role.

Another new result of our study consists in the difference we found between gaze and arrows in driving apparent motion. In particular, in Experiment 1, apparent motion along the vertical directions is seen more often towards the position in which the gaze is pointing, as compared to arrows. In contrast, along the horizontal directions the apparent motion is seen more, or at least in the same proportion, in the direction pointed by the arrow. This was not true in Experiment 2, where no difference was found between gaze and arrows in solving the corresponding problem, although there still was a difference with the line. This result helps to shed some light in the debate on the nature and possible differences between gaze and arrows as central cues, which equally trigger a reflexive-like attentional shift.

In the last decade, several studies have been devoted to investigating possible differences between these two types of directional cues (e.g., [31,40,46,47]). The first studies demonstrating that also central cues could trigger an automatic orienting of attention, were focused on biologically relevant cues, such as eyes and head orientation [32,33,37]. Although afterwards, other investigators have reported that arrow cues can also induce an automatic orienting of attention [30,31,48,49]. Friesen, Ristic & Kingstone [50] showed that only orienting to central arrows can be voluntarily suppressed, suggesting that arrow triggered attention is less automatic than orienting to eyes. This last result seemed to confirm the original distinction between exogenous (i.e., automatic) and endogenous (i.e., voluntary) cues. According to this distinction, arrows should be considered as endogenous, because they are symbolic and therefore require interpretation ([31], but see for example [51], for a different interpretation of arrow as the graphical evolution of the pointing gesture, which, since based on a biological directional cue, retains some of the same properties). Eyes, instead, are different. Eye cues drive automatic orienting because they are resistant to voluntary control since (i) it occurs also when participants know that targets are more likely to appear in the opposite location [32,33,50], and (ii) volitional inhibitory control on saccadic eye movements is reduced when the saccade was previously instructed by a gaze shift toward one of two peripheral targets [52]. However, gaze cues are also different from endogenous cues, given that, in comparison with the automatic orienting induced by peripheral cues, gaze cues result in both a prolonged facilitation and a delayed onset of inhibition processes at the gazed-at location [33,53,54,55]. These peculiarities led some researchers to suggest that gaze is a special attentional cue [33,37,56], and is processed by a special mechanism, as demonstrated by both behavioral and neuropsychological/neuroimaging evidence [57,58,59,60]. The fact that eyes should be considered as “special” would be due to their biological and social significance. This is in analogy with other stimuli, such as biological motion (e.g., [61,62,63,64]), for which a specific mechanism has been suggested as well (e.g., [65], see [66] for a review). Biological motion, as well as eyes (e.g., [67]), could also convey emotions (e.g., [68,69,70]).

Most recently, however, evidence has been accumulated, showing that, when gaze and arrow cues are directly compared within the same group of participants, they are both equally able to induce a very fast orienting of attention when centrally presented [40,46,47,71].

In the present study, we show an important difference between gaze and arrows, as stimuli that can automatically orient visual attention. This difference emerged only in Experiment 1, where only the eye region was presented. This may have induced the perceiver to see the gaze framed within a letter-box shape (i.e., a horizontal bar) against a contrasted background. Given that this was the only difference between Experiment 1 and Experiment 2, we believe that in Experiment 1 a possible ambiguity between the vertical direction (indicated by the gaze itself) and the horizontal direction conveyed by the figural properties of the letter-box shape was solved in favor of the direction of gaze due to its biological and social meaning. In other words, these findings show that, being a biologically and social relevant stimulus, averted gaze has a higher relevance to the perceiver than purely directional cues. When an upward or downward looking gaze is inserted in a horizontal frame, as in Experiment 1, the directional information conveyed by gaze direction (i.e., vertical) is not only processed as more relevant than the one conveyed by the frame (i.e., horizontal), but also induces an attention shift. This results in a higher percentage of responses than that induced by arrows. This finding is particularly meaningful if one considers that, for vertical directions in Experiment 1, a higher proportion of responses toward the direction of the arrow should have been observed, given that no conflict was present for the arrow-stimuli. In other words, the presence of a conflict between the two different possible directions, instead of weakening the effect of gaze direction on the perceived motion, seems to have enhanced gaze relevance by making it a more powerful attentional cue. On the contrary, when no conflict was present, as in Experiment 2, gazes and arrows exerted a similar effect in driving apparent motion in a given direction, indicating that they trigger attentional orienting in a similar way.

This result indicates that the relevance and the strength of the spatial information conveyed by the gaze depends on the context in which the gaze is seen [72]. In other words, orienting to gaze direction takes into account the context associated with the seen gaze (see also [60,73,74,75]). By contrast, the spatial information conveyed by arrows is not affected by the context or task demand [76] and can be processed faster (e.g., [77]). Once the context difference is eliminated (Experiment 2), the main difference between gaze and arrows may rest only on the cortical mechanisms underlying the processing of gaze direction and arrow stimuli (e.g., [60,75]).

Future research on the correspondence problem should test possible conflicts between the direction indicated by the arrow and the one conveyed by the figural properties of a surrounding presentation frame (which has not been tested in the present study). The same holds for the possible additive effect of gaze and figural properties of the presentation frame in the vertical directions. To this end, a new experimental paradigm should be designed in which arrows and gaze should be inserted in different shaped presentation frames (i.e., both horizontal and vertical).

## 5. Conclusions

In conclusion, we found behavioral evidence that stimuli known to automatically orient visual attention, such as gaze direction and arrows, influence motion perception and play a role in solving the correspondence problem, although they are external stimuli [67]. Moreover, the present work shows that the directional information conveyed by these stimuli exerts a stronger effect than that conveyed by the orientation of a line.

Interestingly however, in solving the correspondence problem, the direction indicated by gaze also wins against the direction pointed by the arrows when there is a spatial conflict, suggesting that gaze direction is processed by the perceiver as more informative. Therefore, the present study provides interesting and new ideas for both motion perception and attentional cueing research.
